# Depression and Its Predictors in Adults Receiving Antiretroviral Therapy

**DOI:** 10.7759/cureus.82222

**Published:** 2025-04-14

**Authors:** Azizat A Lebimoyo, Mumtaz Sanni, Mujeedat A Hassan

**Affiliations:** 1 Psychiatry, Lagos State University Teaching Hospital, Lagos, NGA; 2 Medicine, Lincoln County Hospital, Lincoln, GBR; 3 Family Medicine, General Hospital Odan, Lagos, NGA

**Keywords:** anti-retroviral therapy, depression, hiv/aids, mental health, predictors

## Abstract

Background and aim

Depression is a common psychiatric morbidity seen in adults living with HIV/AIDS. Several contextual factors have been implicated in the pathogenesis of depression in HIV-positive adults, these factors could be clinical, treatment-related, or socio-demographic in nature. Due to the debilitating nature of depression and its adverse effect on antiretroviral treatment adherence, HIV-positive adults living with depression are at risk of sub-optimal treatment response and, consequently, poor health outcomes. This suggests that there is a need for more research on depression and its risk factors in HIV-positive adults, as this could inform the promotion of preventive measures against the disorder, as well as early identification and treatment of affected persons. This study aimed to determine the prevalence and predictors of depression among HIV-positive adults receiving antiretroviral therapy.

Methods

This was a cross-sectional study of 257 HIV-positive adults conducted at the Hematology Clinic of the Lagos State University Teaching Hospital, Ikeja, Nigeria. Respondents were selected using a systematic random sampling method. Study instruments used were as follows: the Patient Health Questionnaire (PHQ-9), the four-item Morisky Medication Adherence Scale (MMAS-4), the Multidimensional Perceived Social Support Scale (MPSSS), and a socio-demographic/clinical data questionnaire. Data were analyzed using the EPI-Info statistical analysis software version 7.0 (Atlanta, GA: Centers for Disease Control and Prevention). Univariate, bivariate, and multivariate analyses were done to determine the prevalence and predictors of depression among HIV-positive adults.

Results

The prevalence of depression was 54%, and predictors of depression were being unmarried (n=91, OR=10.9, 95% CI=4.6-26.2, p=0.000), female gender (n=119, OR=5.8, 95% CI=2.2-15.5, p=0.000), low social support (n=57, OR=11.2, 95% CI=4.1-30.8, p=0.000), and unemployment (n=27, OR=0.2, 95% CI=0.1-0.7, p=0.007).

Conclusion

Depression is frequently reported among HIV-positive adults, and its occurrence may be influenced by clinical, social, and demographic-related factors. These variables need to be considered in patient care at antiretroviral clinics in order to achieve a good treatment prognosis. Furthermore, future research may focus on intervention studies on depression in HIV-positive adults, especially in those with increased vulnerability to depression.

## Introduction

For decades, human immunodeficiency virus/acquired immunodeficiency syndrome (HIV/AIDS) has maintained its status as a public health problem due to its major contribution to global morbidity and mortality [1]. With over 40 million people living with HIV/AIDS (PLWHA) globally, Africa appears to be the worst hit region with the HIV/AIDS epidemic, accounting for 60% of the global HIV population, and adults constituting the majority of PLWHA in Africa [2]. Similar to persons living with other chronic diseases, PLWHA have an increased predisposition to psychiatric disorders [2,3]. Depression is the most prevalent psychiatric disorder reported by HIV-positive adults [4,5]. According to the Diagnostic and Statistical Manual of Mental Disorders (Fifth Edition), to diagnose depression, there must be at least five of the following symptoms for a minimum of two week: depressed mood, diminished interest, reduced energy, significant weight loss/gain, change in appetite, insomnia/hypersomnia, feelings of worthlessness, poor concentration, recurrent thoughts of death, suicidal ideations, or suicidal attempt [6].

The relationship between HIV/AIDS and depression is explainable from different perspectives [3]. For instance, depression is a risk factor for HIV/AIDS infection due to impaired decision-making and judgment associated with the disorder, thereby increasing the risk of contracting HIV/AIDS [7]. Depression could arise due to the initial shock of receiving an HIV/AIDS diagnosis, the physical impact of HIV/AIDS, neuropsychiatric manifestations of HIV/AIDS itself, the side effects of antiretroviral therapy (ART), and HIV-related stigma [7].

In Nigeria, the prevalence of depression has been reported as 31% [8], 12.2% [9], 56.7% [10], and 39.1% in different parts of the country [11]. Several socio-demographic and clinical factors have been identified as predictors of depression among HIV-positive adults receiving ART [12,13]. The public health significance of depression in PLWHA is not only limited to its negative impact on mental health but also its link with ART non-adherence and ultimately poor treatment outcomes [14]. Therefore, having a sufficient knowledge of the prevalence and potential predictors of depression in HIV-positive adults may facilitate its prevention, early diagnosis, and treatment. Albeit the existence of a few remarkable mental health studies on PLWHA in Nigeria, there is sporadic data concerning the magnitude and determinants of depression exclusively in HIV-positive adults. Therefore, this study aimed to address this void by exploring the prevalence and predictors of depression among HIV-positive adults receiving antiretroviral therapy (ART) in Lagos, Nigeria. It is hoped that our study findings will encourage the early identification of depression in HIV-positive adults who are at risk of the disorder, as well as result in the prompt management of depression in them.

## Materials and methods

This was a cross-sectional study done between June 2024 and July 2024 among HIV-positive adults receiving ART at the hematology outpatient clinic of the Lagos State University Teaching Hospital (LASUTH), Ikeja, Lagos, Nigeria. Systematic random sampling was used to select 257 participants, this was done by selecting every fifth HIV-positive adult on the daily clinic register as a study participant. Inclusion criteria were as follows: patients aged 18 years and above and those who gave consent for participation were included in this study. Those who had been receiving ART for less than one month were excluded.

Study instruments

Socio-Demographic Questionnaire

A structured self-administered questionnaire was used to obtain socio-demographic data, such as age, gender, marital status, religion, educational status, employment status, and monthly income (questionnaire in appendix).

Clinical Data Questionnaire

A structured questionnaire was created to document clinical information, such as time since HIV/AIDS diagnosis, duration of ART, presence of comorbidities, history of psychoactive substance use, past mental illness, and family history of mental illness (questionnaire in appendix).

Patient Health Questionnaire-9 (PHQ-9)

The PHQ-9 is a self-administered brief questionnaire used to diagnose and assess the severity of depression [15].This instrument has nine items/questions, each item is rated on a Likert scale ranging from 0 to 3, and the respondent is asked to rate how often each symptom occurred over the last two weeks. Total scores range between 0 and 27, severity is rated as follows: 0-4=no depression, 5-9=mild depression, 10-14=moderate depression, 15-19=moderately severe depression, and 20-27=severe depression. The PHQ-9 has been validated for use in Nigeria with a Cronbach’s alpha internal consistency value of 0.90 [16].

Morisky Medication Adherence Scale-four item (MMAS-4)

The MMAS-4 is an instrument that is used to evaluate the level of medication adherence. It consists of four items with a scoring scheme of "yes"=1 and "no"=0, and items are summed to give a range of scores from 0 to 4 [17]. Scores above zero suggest poor medication adherence, while a zero score indicates good medication adherence. The scale has been validated in Nigeria with a Cronbach’s alpha internal consistency value of 0.79 [18].

Multidimensional Perceived Social Support Scale (MPSSS)

The MPSSS is a 12-item self-report scale used to measure perceived social support from family, friends, and significant others [19]. The item questions are scored on a seven-point Likert scale ranging from 1 (very strongly disagree) to 7 (very strongly agree), with high scores indicating higher perceived social support. The scoring system is as follows: 12-35 (low perceived social support), 36-60 (medium perceived social support), and 61-84 (high perceived social support). The MPSSS has been validated for use in Nigeria, having a Cronbach’s alpha internal consistency value of 0.93 [20].

Procedure

Participants were informed about the study purpose, questionnaires were administered, and those identified as having depressive symptoms were referred to the psychiatry unit of the Lagos State University Teaching Hospital for further mental health evaluation and intervention.

Ethical Considerations

Ethical approval was obtained from the Health Research and Ethics Committee of the Lagos State University Teaching Hospital, Ikeja (HREC number: LREC/06/10/2453). Verbal and written informed consent were also obtained from the study participants.

Data Analysis

Data obtained were entered into Microsoft Excel (Redmond, WA: Microsoft Corp.) and analysed with Epi-Info statistical software package version 7 (Atlanta, GA: Centers for Disease Control and Prevention). Univariate, bivariate, and multivariate analyses were carried out accordingly. For the univariate analysis, frequencies and percentages of categorical variables as well as means and standard deviations of continuous variables were determined. For bivariate analysis, the chi-square and Fisher's exact test were used to compare differences between proportions, while the Student's t-test was used for comparison of differences between means. For multivariate analysis, logistic regression was carried out for independent variables that were statistically significant during bivariate analysis. In the logistic regression analysis, the dependent variable was depression, and the socio-demographic covariates were gender, marital status, educational status, monthly income, employment status, and social support. The clinical covariates in the regression analysis were past history of mental illness, ART adherence, and the presence of comorbidities. A p<0.05 was considered statistically significant for all the statistical tests.

## Results

A total of 257 participants were included in the study. All the administered 257 questionnaires were retrieved and analyzed, giving a response rate of 100%.

Socio-demographic and clinical characteristics

The mean age of the sample was 45 (±11.4) years, with most of them in the age group of 50-59 years (n=99, 38.5%). Close to two-thirds had secondary school as their highest level of education (n=143, 55.6%). Also, most of the respondents were self-employed (n=170, 66.2%), and earned less than 100,000 Naira per month (n=227, 88.3%). The majority of the respondents contracted HIV through unknown means (n=158, 61.5%). Almost half of the respondents (n=118, 46%) had been diagnosed with HIV/AIDS over a year ago and had poor ART adherence (n=107, 41.6%), but most (n=170, 66.1%) had been receiving ART for less than a year. A minority of them had a past history of mental illness (n=38, 14.8%), and a family history of mental illness (n=52, 20.2%). However, a little over one-third of the sample had some form of comorbidity (n=84, 32.7%) (Table 1).

**Table 1:** Socio-demographic and clinical characteristics of the respondents. SD: standard deviation

Variables	Frequency (%)
Age (years)	
20-29	30 (11.7)
30-39	41 (16.0)
40-49	70 (27.2)
50-59	99 (38.5)
60-69	17 (6.6)
Mean±SD = 45±11.4	N/A
Gender	
Male	60 (23.3)
Female	197 (76.7)
Religion	
Islam	62 (24.1)
Christian	195 (75.9)
Educational status	
Primary	46 (17.9)
Secondary	143 (55.6)
Tertiary	68 (26.5)
Employment status	
Employed	46 (17.9)
Self-employed	170 (66.2)
Unemployed	41 (15.9)
Marital status	
Single	34 (13.2)
Married	142 (55.3)
Divorced	14 (5.5)
Separated	21 (8.1)
Widowed	46 (17.9)
Monthly income (Naira)	
<100,000	227 (88.3)
100,000-150,000	23 (9.0)
>150,000	7 (2.7)
Mean±SD = 42,385±39,933	N/A
Social support (score)	
Low (12-35)	68 (26.5)
Medium (36-60)	105 (40.8)
High (61-84)	84 (32.7)
Mean±SD = 46±20	N/A
Time since HIV diagnosis (months)	
<6	62 (24.1)
6-12	77 (29.9)
>12	118 (45.9)
Mean±SD = 10.9±7.1	N/A
Duration of ART (months)	
<6	73 (28.4)
6-12	97 (37.7)
>12	87 (33.8)
Mean±SD = 10.6±6.7	N/A
ART adherence (score)	
Poor (1-4)	107 (41.6)
Good (0)	150 (58.4)
Mean±SD = 2±1	N/A
Route of HIV transmission	
Perinatal	0 (0.0)
Blood transfusion	28 (10.9)
Contact with sharps	28 (10.9)
Sexual	43 (16.7)
Unknown	158 (61.5)
Presence of comorbidities	
None	173 (67.3)
Tuberculosis	32 (12.5)
Diabetes mellitus	19 (7.4)
Hypertension	8 (3.1)
Cancer	9 (3.5)
Fungal infection	16 (6.2)
Past history of mental illness	
No	219 (85.2)
Yes	38 (14.8)
Family history of mental illness	
No	205 (79.8)
Yes	52 (20.2)
Depression (score)	
None (0-4)	116 (46)
Mild (5-9)	34 (13)
Moderate (10-14)	50 (20)
Moderately severe (15-19)	29 (11)
Severe (20-27)	28 (10)
Mean±SD = 9.3±8.4	N/A

Prevalence of depression

The PHQ-9 was used to screen for and determine the severity of depression, and it has a total score ranging between 0 and 27. The participants were asked to rate how often each symptom in the questionnaire occurred over the last two weeks. The total scores for the participants were interpreted as follows: 0-4=no depression, 5-9=mild depression, 10-14=moderate depression, 15-19=moderately severe depression, and 20-27=severe depression. Our findings showed that the prevalence of depression among the respondents was 54%; however, different levels of severity were found. To be specific, rates of 13%, 20%, 11%, and 10% were reported for mild, moderate, moderately severe, and severe depression, respectively (Table 1).

Predictors of depression

On bivariate analysis, socio-demographic variables such as age, monthly income, gender, marital status, educational status, employment status, and social support had their associations with depression analyzed. It was also revealed that the time since diagnosis and duration on ART had a notable correlation with depression. Other clinical variables, such as ART adherence, presence of comorbidities, past history of mental illness, and family history of mental illness, had a significant association with depression (Table 2).

**Table 2:** Bivariate analysis showing factors associated with depression. SD: standard deviation; n: frequency

Variable	Depression absent, n (%)	Depression present, n (%)	Depression absent (mean±SD)	Depression present (mean±SD)	Chi-square value	t-Value	p-Value
Age (years)	N/A	N/A	45.9±10.4	45.1±12.2	N/A	0.58	0.562
Monthly income (Naira)	N/A	N/A	52,397±48,143	34,149±29,337	N/A	3.74	0.000
Time since HIV diagnosis (months)	N/A	N/A	11.8±7.1	10.3±7.1	N/A	1.68	0.094
Duration of ART (months)	N/A	N/A	9.9±6.3	11.2±6.9	N/A	1.63	0.104
Gender							
Male	38 (63.3)	22 (36.7)	N/A	N/A	9.53	N/A	0.002
Female	78 (39.6)	119 (60.4)					
Marital status							
Single	10 (29.4)	24 (70.6)	N/A	N/A	N/A	N/A	0.000 (Fisher’s exact)
Married	92 (64.8)	50 (35.2)					
Divorced	6 (42.9)	8 (57.1)					
Separated	0 (0.0)	21 (100.0)					
Widowed	8 (17.4)	38 (82.6)					
Educational status							
Primary	5 (10.9)	41 (89.1)	N/A	N/A	26.7	N/A	0.000
Secondary	74 (51.8)	69 (48.2)					
Tertiary	37 (54.4)	31 (45.6)					
Employment status							
Employed	31 (67.4)	15 (32.6)	N/A	N/A	11.9	N/A	0.003
Self-employed	71 (41.8)	99 (58.2)					
Unemployed	14 (34.2)	27 (65.8)					
Social support							
Low	11 (16.2)	57 (83.8)	N/A	N/A	31.5	N/A	0.000
Medium	57 (54.3)	48 (45.7)					
High	48 (57.1)	36 (42.9)					
ART adherence							
Good	83 (55.3)	67 (45.7)	N/A	N/A	14.21	N/A	0.000
Poor	33 (30.8)	74 (69.2)					
Presence of comorbidities							
No	87 (50.3)	86 (49.7)	N/A	N/A	5.06	N/A	0.025
Yes	29 (34.5)	55 (65.5)					
Past history of mental illness							
No	111 (50.7)	108 (49.3)	N/A	N/A	16.9	N/A	0.000
Yes	5 (13.2)	33 (86.8)					
Family history of mental illness							
No	90 (43.9)	115 (56.1)	N/A	N/A	0.4	N/A	0.527
Yes	26 (50.0)	26 (50.0)					
Substance use							
No	101 (46.9)	114 (53.0)	N/A	N/A	1.37	N/A	0.241
Yes	15 (35.7)	27 (64.3)					

Multivariate analysis showed that depression was significantly influenced by female gender (OR=5.8, 95% CI=2.2-15.5, p=0.000), being unmarried (OR=10.9, 95% CI=4.6-26.2, p=0.000), unemployment (OR=0.2, 95% CI=0.1-0.7, p=0.007), and low social support (OR=11.2, 95% CI=4.1-30.8, p=0.000) (Table 3).

**Table 3:** Results of logistic regression model for predictors of depression. ART: antiretroviral therapy

Variable	Odds ratio	95% Confidence interval (lower limit)	95% Confidence interval (upper limit)	p-Value
Female gender	5.79	2.17	15.46	0.000
Tertiary education	0.87	0.41	1.82	0.711
Unemployment	0.23	0.08	0.67	0.007
Monthly income of <100,000 Naira	1.32	0.45	3.85	0.607
Being unmarried	10.98	4.62	26.15	0.000
Low social support	11.22	4.08	30.84	0.000
Past history of mental illness	3.37	1.02	11.14	0.046
Poor ART adherence	0.89	0.42	1.86	0.751
Presence of comorbidities	1.55	0.75	3.17	0.229

## Discussion

A prevalence of 54% for depression was found among HIV-positive adults, suggesting that approximately one in two HIV-positive adults experienced depression. The prevalence of depression found in this study is comparable with 56% and 56.7% recorded in past hospital-based studies in Nigeria and Turkey, respectively [10,21]. However, the prevalence of 54% was higher than 36% and 41% found in earlier research from South Korea and Romania, respectively [22,23]. The disparities in prevalence between the present study and the previous ones may be due to the studies' methodological differences, such as variations in sample size and sampling technique. Furthermore, cultural and socio-economic differences between HIV-positive adults across the country may have also contributed to the varying prevalence rates. The high prevalence of depression in HIV-positive adults signifies the need for routine screening for depression in them by their physicians at every ART clinic appointment. This would ensure the timely detection and management of depression in these patients.

Female HIV-positive adults were six times more likely to have depression than male HIV-positive adults. This finding is in line with previous research, which found a positive relationship between female gender and depression among adults living with HIV/AIDS [8,24].Gender differences in depression are well-established in the general population, with female gender recognized as a vulnerability factor. A similar pattern appears to hold true among people living with HIV/AIDS (PLWHA) [25,26]. Females play multiple roles in the home, workplace, and society at large, undoubtedly, this is bound to be overwhelming. Notably, females tend to suffer from depression more than men due to their greater exposure to socio-economic disadvantages, such as increased risk of gender-based violence, lack of formal education, inadequate financial empowerment, power imbalance in romantic relationships, and limited autonomy [27].

For a female adult living with HIV, the above-listed stressors could be exacerbated by the physical impact of HIV/AIDS and ART, coupled with the additional burden of hormonal changes associated with menstruation, pregnancy, childbirth, and menopause. Having to deal with all these psychosocial stressors may herald the onset, and subsequently, perpetuate the course of depression in female HIV-positive adults. This indicates that there must be a high index of suspicion for depression in female adults attending ART clinics.

Unmarried HIV-positive adults were more prone to depression in comparison with their married counterparts. This finding echoes reports from prior research in which being unmarried was significantly linked to depression among HIV-positive adults [28,29]. This observation could be due to the possibility that unmarried people, especially those divorced or widowed, may experience more challenges in the form of insufficient moral support, inability to cope with work pressure, and the stress of single parenthood [30].

Beyond this, for an adult living with HIV/AIDS, being unmarried may lead to increased stress, financial insecurity, societal pressure to conform to marital norms, as well as stigmatization and discrimination from their families and the society at large [30]. Unmarried HIV-positive adults may also face the additional challenge of lacking spousal support and having no partner with whom to share their health concerns. These factors can contribute to feelings of loneliness, helplessness, and hopelessness, which may ultimately lead to depression [31].

Low social support was associated with an 11-fold increased risk of depression among adults living with HIV/AIDS. This observation is consistent with what has been documented in earlier studies [8,32]. Similar findings were made in a study in which a significant relationship was found between low social support and the occurrence of depression among Somali HIV-positive adults [28]. Adults living with HIV/AIDS are at risk of low social support due to rejection, loss of relationships due to their HIV status, and a reduced sense of belonging within society [33]. Low social support could lead to feelings of hopelessness, self-esteem issues, reduced confidence, anxiety, and ART non-adherence, which may cause further immunosuppression, and eventually, depression [34].

Adults living with HIV/AIDS, who were also unemployed, had an elevated risk of depression in comparison to those who had some form of employment. Similar reports have been made from past research involving HIV-positive adults [31,35]. Bearing in mind that HIV/AIDS is associated with immunosuppression, thereby increasing susceptibility to a variety of infectious diseases and other ailments, a regular, healthy, and nutritious diet is required to improve immunity and overall wellness. However, achieving this could be a challenge for unemployed HIV-positive adults, as they may lack the financial wherewithal to procure the necessary foods and nutritional supplements. Consequently, unemployed HIV-positive adults may experience worsened physical health states, and eventually, marked emotional distress in the form of depression. In addition, unemployed HIV-positive adults may have to depend on others for financial support, this may be demoralizing, bring about feelings of being a burden to family and friends, diminished self-worth, and social withdrawal, all of which may potentiate their risk for psychiatric morbidity such as depression [31,35].

Limitations

The use of self-reporting questionnaires increased the likelihood of selection of socially desirable responses by the participants. In addition, being a cross-sectional study, a causal relationship could not be established between depression and the identified predictors. Prospective longitudinal studies on the trajectory of depression and its predictors in HIV-positive adults may provide valuable insights in the future.

Policy and clinical implications

There is a need for the formulation of policies that would foster increased support for PLWHA, most especially, adults who are unemployed, female, and lack sufficient social support. This may be achieved via the creation of nationwide supportive employment opportunities, community support programs, and other welfare packages for vulnerable HIV-positive adults. Regular screening for depression in HIV-positive adults by healthcare workers in ART clinics is a necessity, as this would enhance timely diagnosis and treatment.

Future directions

Intervention studies concerning the impact of psychotherapy on depression in HIV-positive adults in Nigeria could be considered for future research, as there are sparse studies concerning this subject matter within this environment.

## Conclusions

Adults living with HIV/AIDS are susceptible to depression, and risk factors for depression should be considered in patient care at ART clinics across the country. Most importantly, there is a need for improved social support for adults living with HIV/AIDS. This may come in the form of peer support groups, supportive community programmes, and mental health services. Hopefully, this will lead to an increase in the level of social support provided to these patients, thereby lessening their susceptibility to depression.

## Figures and Tables

**Figure 1:**
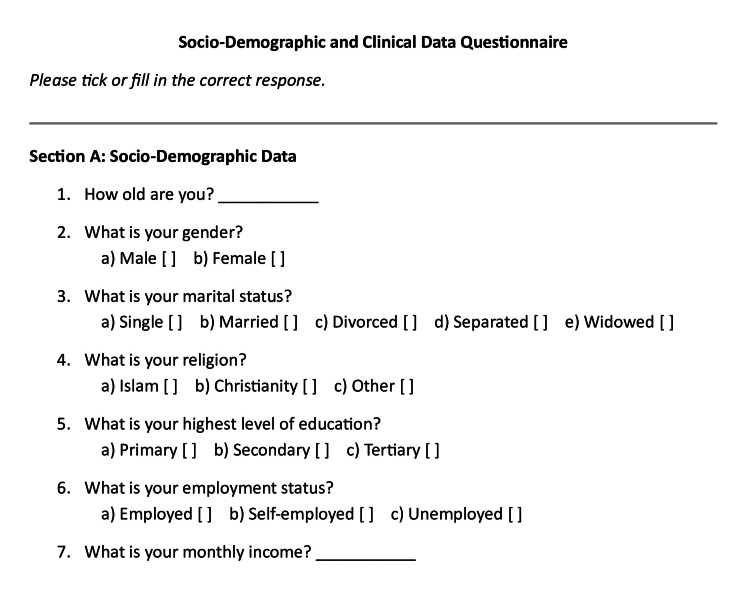
Socio-demographic and clinical data questionnaire: Section A.

**Figure 2:**
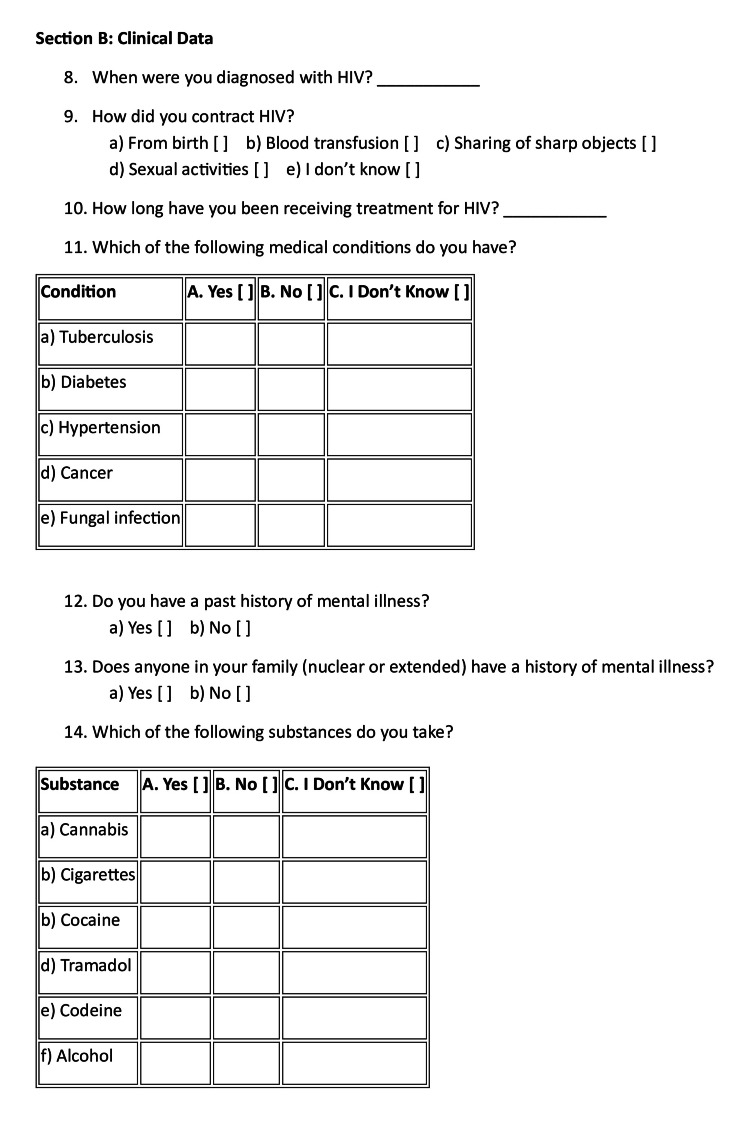
Socio-demographic and clinical data questionnaire: Section B.
